# Erythrokeratoderma variabilis (EKV) – First Nepalese case documenting *GJB3* mutation

**DOI:** 10.1002/ski2.63

**Published:** 2021-08-17

**Authors:** M. Shah, S. Baral, R. C. Adhikari

**Affiliations:** ^1^ Department of Dermatology Anandaban Hospital The Leprosy Mission Nepal Lalitpur Nepal; ^2^ Department of Pathology Anandaban Hospital The Leprosy Mission Nepal Lalitpur Nepal; ^3^ Department of Pathology Institute of Medicine Maharajgunj Medical Campus Tribhuvan University Kathmandu Nepal

## Abstract

Erythrokeratoderma Variabilis (EKV) is a rare genodermatosis, characterized by variable erythematous and hyperkeratotic skin lesions. It is most often transmitted in autosomal dominant manner (AD). Casual mutations were found in the *GJB3* and *GJB4* genes encoding connexins 31 and 30.3, respectively. We report a 7‐year‐old girl with multiple dusky red and brown skin lesions on face, buttock, both arms and legs. This diagnosis was made on the basis of clinical and histological findings and further genetic analysis detected a G > C transition at position 125 of the coding sequence, which replaces arginine with a proline at residue 42 of the protein (R42P). Here, we report a first case of Nepalese patient with EKV resulting from the *GJB3* mutation.

## Graphical abstract

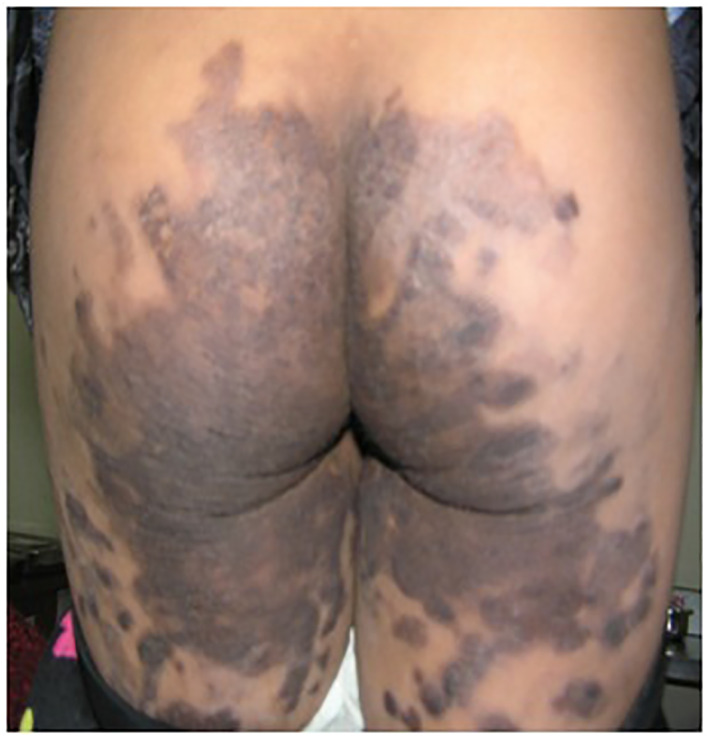

1

**What's already known about this topic?**

- Erythrokeratoderma variabilis is a genetic disease with various gap junction protein mutations. The disease distribution in different communities is not known.

**What does this study add?**

- This is the first case reported from Nepal, which shows that the disease is present in different communities. Overall genodermatosis is considered rare in Nepal which may be due to unavailability of genetic testing. This type of cases stimulates dermatology community to consider genetic testing in patient with favourable clinical presentation.

## 2 INTRODUCTION

Erythrokeratoderma variabilis (EKV) is a rare genetic disorder of keratinization. EKV is associated with mutation in the genes *GJB3* ^1^ or *GJB4* ^2^ or *GJA1* ^3^ which encodes gap junction proteins connexin 31, connexin 30.03 and connexin 43, respectively. EKV is usually inherited in an autosomal dominant fashion, with variable penetrance, so there is considerable inter‐ and intrafamilial variability in severity of the skin diseases. Skin lesions are characterized by the coexistence of fixed brownish red hyperkeratotic plaques mainly on the extensor surfaces of the limbs, and transient erythematous macules that may be of unusual shape. Clinical signs and symptoms usually present at birth or begin during infancy.^4^

To the best of our knowledge, no case of EKV with genetic analysis has been documented from Nepal. We report first case of EKV from Nepal with *GJB3* mutation.

### 2.1 Case report

**FIGURE 1:**
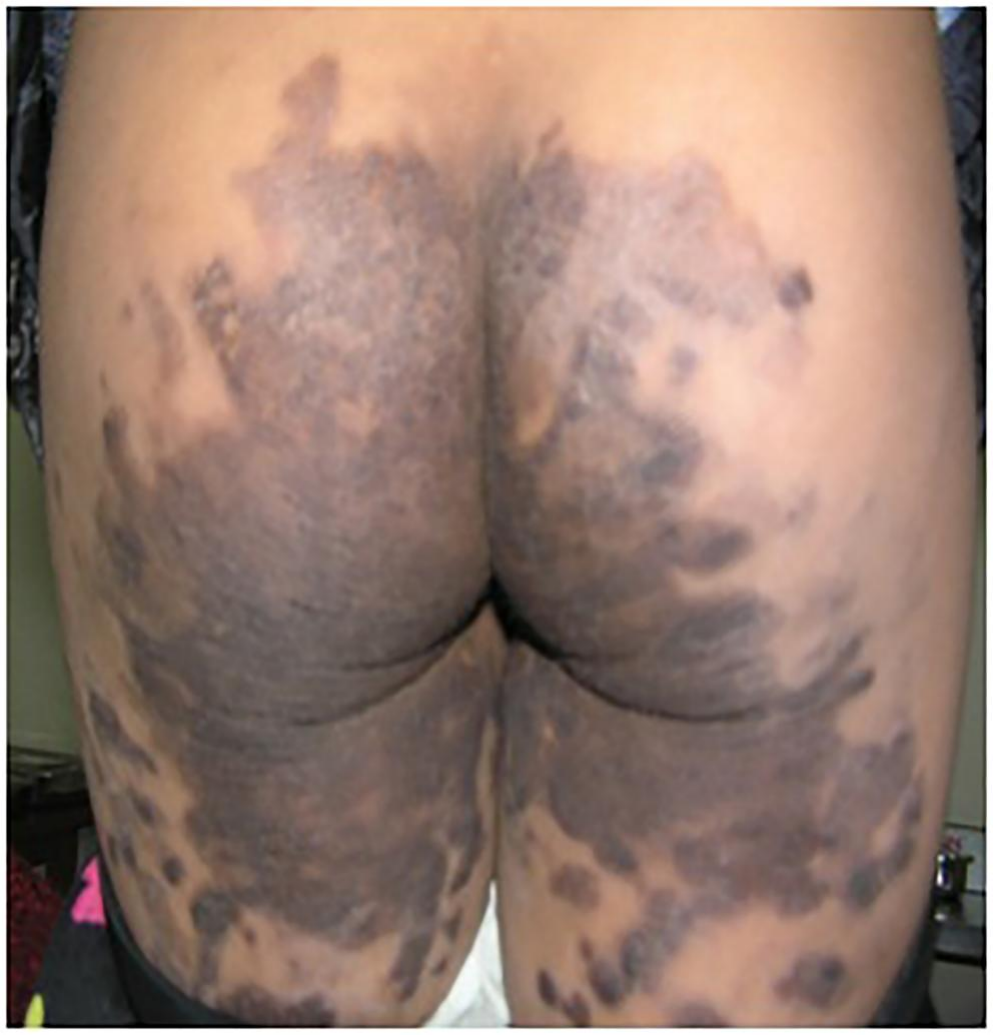
Hyperkeratotic plaque on buttock

**FIGURE 2:**
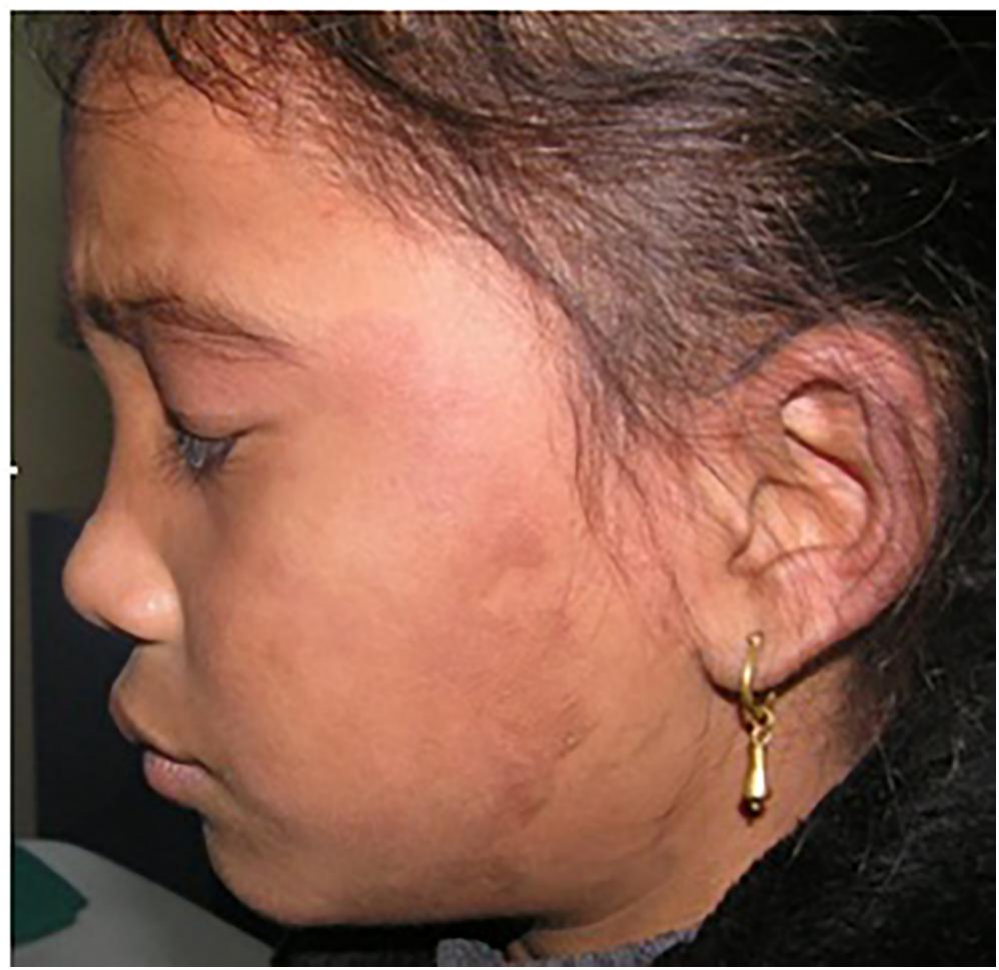
Dusky erythematous macular lesion on face

A 7‐year‐old girl presented with multiple well‐defined, irregular, variably sized brownish plaques with fine scales over the arms, legs, ankles and buttocks (Figure 1) and dusky red macules on the face (Figure 2). The lesions first appeared at the age of 5 months starting at the malar area as reddish lesion which gradually progress to involve other parts of the body. Initially the lesions start as red macules, which turned to brown black plaques over the months. The lesions worsened during winter and improved in summer. The palms, soles, scalp, hair, nails, teeth and mucosa were normal.

She was a single child and there was no history of similar skin problems in the family. There were no features suggestive of other systemic illness. Laboratory tests including complete blood count, liver and kidney function tests and urine analysis were within normal limits.

Skin biopsy from posterior aspect of left leg showed features compatible with erythrokeratoderma variabilis. The findings were orthohyperkeratosis, acanthosis with focally broadened granular layer. Upper dermis showed melanin pigment incontinence positively stained with Masson‐Fontana stain, and perivascular lymphohistiocytic infiltrate.

**FIGURE 3:**
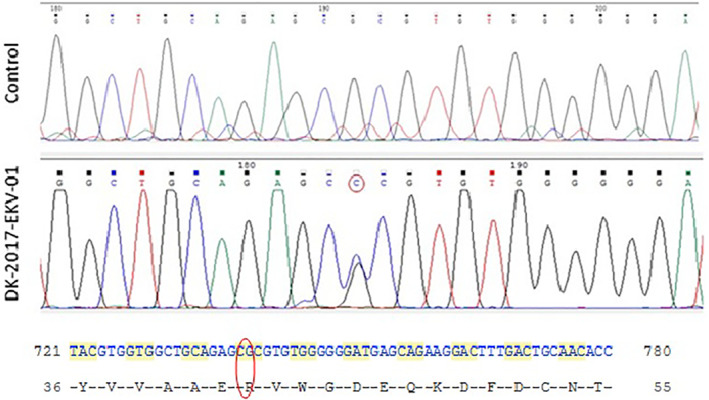
Genetic analysis showing translation mutation of *GJB3* gene with the mutated residue circled in red

Based on clinical and histological findings, the diagnosis of EKV was established and genetic analysis was performed for *GJB3* and *GJB4* gene mutation. Genomic DNA was extracted from blood and the coding exons of *GJB3* and *GJB4* were amplified by PCR and sequenced. Sequence analysis revealed patient was heterozygous for the single nucleotide mutation c.125G > C in exon 2 of the *GJB3* gene (encoding connexin 31), predicted to cause a missense change in an arginine to proline in codon 42 (p.Arg42Pro) (Figure 3). No disease‐causing mutations were identified in *GJB4*.

Parents were counselled about the nature of the disease and the patient was managed symptomatically with emollients.

## 3 DISCUSSION

EKV has variability in both genotype and phenotype. So individual patient may not have all identified genetic mutations and clinical manifestations. Gap junction protein (connexin) mutations play a major role in disease pathogenesis of EKV. In approximately half of the patients with EKV, the causal genetic mutation has been mapped to chromosome1p34–p35.1. This locus harbours the genes *GJB3* and *GJB4* encoding Cx 31 and 30.3, respectively.^5^

Connexin (Cx) forms the transmembrane channels between the keratinocytes to permit rapid exchange of ions and small molecules for the maintenance of tissue homeostasis, growth control, development and synchronized response of cells to stimuli.^6^ In skin Cx31 and Cx30.3 are expressed in the stratum granulosum of the epidermis with a suggested role in late keratinocytes differentiation.^4^ At the molecular level, a mutation in Cx30.3 or 31 disrupts protein transport and intercellular communication. Recent evidence in EKV suggests that accumulation of mutant proteins causes the unfolded protein response.^7^ The molecular action of Cx31 pathogenic mutants remains largely elusive. It has been shown that expression of EKV pathogenic mutant Cx31R42P induces cell death with necrotic characteristics. The Cx31R42P active hemichannels are likely resulted by an ER‐stress‐induced reactive oxygen species (ROS) overproduction.^8^

However, not all clinically diagnosed individuals with EKV harbour *GJB3* or *GJB4* or *GJA1*disease‐associated mutations. Even in the same gene there has been associated different point mutations causing different missense changes Table 1.

**TABLE 1:** Different genes and specific mutations found in EKV

Gene	Nucleotide	Codon	Reference
*GJB3*	125G/C	R42P	^9^, ^10^
*GJB3*	34G/C	G12R	^1^
*GJB3*	34G/A	G12D	^1^
*GJB3*	409T/C	F137L	^2^
*GJB4*	411C/A	F137L	^2^
*GJB4*	35 G/A	G12D	^2^
*GJB4*	253 A/C	T85P	^2^
*GJB4*	64 G/A	R22H	^2^
*GJB4*	409T/C	F137L	^2^
*GJB4*	566T/A	F189Y	^2^

Abbreviation: EKV, Erythrokeratoderma variabilis.

Genetic mutation similar to our case that is single nucleotide mutation c.125G > C in exon 2 of the *GJB3* gene causing missense change in an arginine to proline in codon 42 (p.Arg42Pro), was identified separately in six Italian cases, three of each in one pedigree by Wilgoss^9^ and Richard.^10^ These six cases have clinical features of palmoplanter keratoderma (PPK), localized hyperkeratosis and transient erythematous patches, which were similar to our case except for PPK. Palmoplanter keratoderma has been identified in almost 50% of patients with EKV. Transient erythematous patches and stable hyperkeratotic plaques are present in almost all the patients in both *GJB3* (Cx31) and *GJB4* (Cx30.3) mutations.^2^, ^9^, ^10^ Erythema or hyperkeratosis is often triggered by sudden temperature change, emotional stress, mechanical friction, and sun exposure.^2^, ^10^ Some of the other features that has been found in other EKV patients but not in our patient were hypertrichosis,^2^ icthyosis hysterix.^10^

Histopathological features of EKV is not specific to the disease but some consistent features in hyperkeratotic skin lesions are papillomatosis, acanthosis, hypergranulosis and compact orthohyperkeratosis or parakeratosis and follicular plugging.

Treatment of EKV is symptomatic, as none of the treatment modalities are curative. Treatment depends on the severity and extent of the hyperkeratosis. Mild forms can be treated with emollients, topical keratolytics and topical retinoids. Parents should be counselled about the prognosis and regular follow‐up.

Due to rarity of the disease, EKV can often go under‐diagnosed or misdiagnosed for years. So this case report will help the physicians to keep EKV in differential diagnosis.

## CONFLICTS OF INTEREST

None to declare.

## AUTHOR CONTRIBUTIONS

**M. Shah:** Conceptualization; Formal analysis; Funding acquisition; Investigation; Methodology; Resources; Supervision; Writing – original draft; Writing – review & editing. **S. Baral:** Conceptualization; Formal analysis; Methodology; Software; Validation; Writing – original draft; Writing – review & editing. **R. C. Adhikari:** Formal analysis; Supervision; Writing – original draft.
